# P-192. Detection of Sequelae from Acute Meningitis during Clinical Examination by a Healthcare Provider: A Global Systematic Review and Meta-Analysis

**DOI:** 10.1093/ofid/ofaf695.415

**Published:** 2026-01-11

**Authors:** Luisa F Alviz, Carla Y Kim, Lauren E Monette, Caroline E Harrer, Ana C Benevides-Tadinac, Jackson A Roberts, Francisco J Varela, Soonmyung A Hwang, Blen M Gebresilassie, Pilar Balcarce, Manya Prasad, John Usseglio, Kiran T Thakur

**Affiliations:** Columbia University, Bogotá, Distrito Capital de Bogota, Colombia; Columbia university, New York, New York; Columbia University, Bogotá, Distrito Capital de Bogota, Colombia; The Rockefeller University, New York, New York; University of South Carolina, West Columbia, South Carolina; Columbia University, Bogotá, Distrito Capital de Bogota, Colombia; Fleni institute, Buenos Aires, Buenos Aires, Argentina; Icahn School of Medicine at Mount Sinai, New York, New York; Columbia University, Bogotá, Distrito Capital de Bogota, Colombia; Fleni institute, Buenos Aires, Buenos Aires, Argentina; All India Institute of Medical Sciences, New Dehli, Delhi, India; Columbia University, Bogotá, Distrito Capital de Bogota, Colombia; Columbia University Irving Medical Center, Orangeburg, NY

## Abstract

**Background:**

Neurological sequelae from acute meningitis are estimated to affect over 30% of survivors worldwide, although it is underreported, due to inadequate follow-up and diagnostic challenges. The aim of this review is to identify the most appropriate timepoints for detecting meningitis-associated sequelae.

**Methods:**

A literature review was conducted in three databases. Studies documenting the time frame at which sequelae were detected after an acute episode of meningitis were included. Descriptive analysis and meta-analysis of pooled prevalence for outcome were performed, with subgroup analysis per timepoint of healthcare assessment.

**Results:**

Eighty-nine studies met inclusion criteria, reporting 9,311 adult and 18,658 pediatric meningitis cases. Among adults, the most frequent sequelae were hearing loss, followed by focal neurological deficits, psychological, neurocognitive impairments, seizures, hydrocephalus, and speech, and vision impairments. While more were assessed before discharge (5,270 vs. 2,711), the proportion of sequelae diagnoses was higher post-discharge. The pooled prevalence of sequelae was 24.8% (95% CI 20.5–29.2%) at discharge, compared to 41.5% (95% CI 25.7–57.3%) within three months and 31.9% (95% CI 18.5–45.3%) beyond three months post-discharge. In children, the most common sequelae were hearing loss, followed by focal neurological deficits, seizures, neurocognitive and neurodevelopmental impairments. More were assessed post-discharge (8,298 vs. 7,180), with a higher pooled prevalence of sequelae diagnoses post-discharge. At discharge, the pooled prevalence of sequelae was 28.9% (95% CI 20.8–37%), compared to 29.9% (95% CI 19–40.8%) within three months and 38.2% (95% CI 30.3–46.1%) beyond three months after discharge.

**Conclusion:**

Meningitis-related sequelae significantly impact quality of life, though the recommended timeline of sequelae detection is not clear. This review highlights the need for standardized assessment guidelines to ensure timely diagnosis. As the development and course of sequelae varies among patients and age groups, monitoring should begin at discharge and follow-up should also be prioritized as they could still identify undetected sequelae.

**Disclosures:**

Kiran T. Thakur, MD FAAN, Center for Disease Control: Grant/Research Support|Delve Bio: Advisor/Consultant|World Health Organization: Advisor/Consultant

